# The efficacy and safety of quinagolide in hyperprolactinemia treatment: A systematic review and meta-analysis

**DOI:** 10.3389/fendo.2023.1027905

**Published:** 2023-01-24

**Authors:** Yanyang Zeng, Qingliang Huang, Yunzhi Zou, Jiacong Tan, Wu Zhou, Meihua Li

**Affiliations:** ^1^ Department of Neurosurgery, The First Affiliated Hospital of Nanchang University, Nanchang, Jiangxi, China; ^2^ Department of Neurosurgery, The Fifth Affiliated Hospital of Nanchang University (Fu Zhou First People’s Hospital of Jiangxi Province), Fuzhou, Jiangxi, China; ^3^ College of Medical, Nanchang University, Nanchang, Jiangxi, China

**Keywords:** hyperprolactinemia, prolactinomas, dopamine agonist, quinagolide, meta-analysis, cabergoline, bromocriptine, efficacy

## Abstract

**Purpose:**

Three dopamine agonists [bromocriptine, cabergoline, and quinagolide (CV)] have been used for hyperprolactinemia treatment for decades. Several studies have reviewed the efficacy and safety of bromocriptine and cabergoline. However, no systematic review or meta-analysis has discussed the efficacy and safety of CV in hyperprolactinemia and prolactinoma treatment.

**Methods:**

Five medical databases (PubMed, Web of Science, Embase, Scopus, and Cochrane Library) were searched up to 9 May 2022 to identify studies related to CV and hyperprolactinemia. A meta-analysis was implemented by using a forest plot, funnel plot, sensitivity analysis, meta-regression, and Egger’s test *via* software R 4.0 and STATA 12.

**Results:**

A total of 1,211 studies were retrieved from the five medical databases, and 33 studies consisting of 827 patients were finally included in the analysis. The pooled proportions of patients with prolactin concentration normalization and tumor reduction (>50%) under CV treatment were 69% and 20%, respectively, with 95% confidence intervals of 61%–76% and 15%–28%, respectively. The pooled proportion of adverse effects was 13%, with a 95% confidence interval of 11%–16%.

**Conclusion:**

Our study showed that CV is not less effective than cabergoline and bromocriptine in treating hyperprolactinemia, and the side effects were not significant. Hence, this drug could be considered an alternative first-line or rescue treatment in treating hyperprolactinemia in the future.

**Systematic review registration:**

https://www.crd.york.ac.uk/PROSPERO, identifier CRD42022347750.

## 1 Introduction

The synthesis and secretion of prolactin (PRL) are suppressed through the hypothalamic dopamine system (1). Disorders of the hypothalamus usually induce high serum PRL concentrations that subsequently evolve into hyperprolactinemia. The most common cause of hyperprolactinemia is prolactinoma, which is one of the most common pituitary adenomas (2). Patients with hyperprolactinemia can present with bone loss, decreased spinal bone density (3), amenorrhea, galactorrhea, and decreased libido (4). Prolactinoma not only includes the above complications but also may lead to headaches and visual field defects caused by tumor compression or even death due to tumor bleeding. The first-line treatment of hyperprolactinemia and prolactinomas involves the use of dopamine agonists (DAs), the most common of which are cabergoline (CAB) and bromocriptine (BRC), whereas quinagolide (CV) is mainly used in Europe. However, several systematic reviews and meta-analyses have indicated that the remission rate under CAB and BRC treatment is not satisfactory, which is mainly due to recurrence (30%–80%), resistance, and intolerance (5–8).

Unlike CAB and BRC, which are ergot-like DAs, CV is a non-ergot-like DA (9). It may have the potential to overcome the resistance and intolerance to CAB and BRC. In addition, in the study by Colao et al. (10), CV was shown to be more effective than BRC in the implantation inhibition test and lactation inhibition test. Moreover, no systematic review or meta-analysis has discussed the efficacy and safety of CV in hyperprolactinemia and prolactinoma treatment, whereas many clinical trials have explored this concept. Hence, we performed this systematic review and meta-analysis to assess the efficacy and safety of CV in treating hyperprolactinemia and prolactinomas.

## 2 Methods

### 2.1 Study registration

This study is registered in PROSPERO (International Prospective Register of Systematic Reviews) (CRD42022347750). The registration information is shown in the following link: https://www.crd.york.ac.uk/PROSPERO/display_record.php?RecordID=347750.

### 2.2 Information sources

Literature retrieval was performed by using five databases, including PubMed, Web of Science, Embase, Scopus, and Cochrane Library. There was no limitation on the published language. Retrieval was restricted by studies published before 9 May 2022.

### 2.3 Search

The search term was “quinagolide and (prolactinoma or hyperprolactinemia).”

### 2.4 Study selection

Retrieval studies were loaded into the Reference management software NoteExpress 3.2.0.7276 (AegeanSoft Corporation). Duplicate studies among different databases were removed. In addition, preliminary screening was performed based on the title, abstract, and keywords. The remaining studies were subsequently screened based on inclusion and exclusion criteria under full-text reading.

Both groups were screened simultaneously. YYZ and JCT were in Group A. QLH and WZ were in Group B. The two groups were screened separately by first reviewing the titles, abstracts, and keywords. Disagreements were resolved by discussion. Afterward, full texts were screened. Any objections were resolved *via* discussions with more experienced researchers (MHL and YZZ).

The inclusion criteria were as follows:

i. Participants were hyperprolactinemia or prolactinoma patients;
ii. CV was the only intervention or one of the interventions;
iii. The proportion of patients with normalized PRL concentrations or tumor reductions (>50%) is reported or can be calculated;
iv. If there were duplicated cohorts, the largest cohort was included in the analysis.
The exclusion criteria were as follows:
i. The normal reference values of PRL were not reported;
ii. The dosage of DAs was not reported;
iii. The smaller cohorts were removed if duplicated cohorts were presented;
iv. Studies had high heterogeneity;
v. Acromegaly, plurihormonal pituitary adenomas, hyperprolactinemia due to drug use, and renal failure.

### 2.5 Data collection process

All of the authors related to the data collection process were divided into two groups. YYZ and JCT were in Group A. QLH and WZ were in Group B. Group A and Group B separately extracted the related data into an Excel table. Disagreements were resolved by discussions within the two groups. Any disagreements without consensus were discussed with experimental researchers (MHL and YZZ).

### 2.6 Data items

We extracted the following data: year of publication, research area, study type, cause of hyperprolactinemia, drug used, numbers and ages of the included patients, sex information, methods of tumor detection, methods of serum PRL concentration measurement, initial serum PRL concentrations of the included patients, information on tumor reduction, and information on PRL concentration normalization.

### 2.7 Summary measures

The proportion of normalized PRL concentrations and tumor reductions (>50%) under DA treatment were the outcome indicators in the individual studies. The pooled proportions, risk ratios (RRs), and 95% confidence intervals (CIs) were the effect sizes in this systematic review and meta-analysis.

### 2.8 Synthesis of results

Two main units of serum PRL concentration (ng/ml and mIU/L) were used in the included studies. In our study, ng/ml (the conversion factor is 30) was used as the serum PRL concentration (8). Prolactinoma is classified according to size, and the diameter is shown in mm. Microprolactinoma is less than 1 mm, and macroprolactinoma is 1 mm or more (11). The proportion of normalized PRL concentrations and tumor reductions (>50%) under DA treatment were calculated and pooled. These values are presented by using pooled proportions and RRs with 95% CIs. In terms of heterogeneity among the included studies, we performed the I^2^ test and χ^2^ statistic test. Significant heterogeneity was indicated if P < 0.1 (χ^2^ statistic) or I^2^ > 50% (I^2^ test), and a random-effects model was used for the meta-analysis; otherwise, a fixed-effects model was performed. Heterogeneity was analyzed *via* stratified analysis, sensitivity analysis, and meta-regression. In the sensitivity analysis, each study was omitted one by one with a changed effect size. In the stratified analysis and meta-regression, the included studies were divided based on treatment line and the size of the prolactinomas before DA treatment. The source of heterogeneity was indicated according to a statistically significant P value (P < 0.05). Publication bias was evaluated *via* funnel plots and the Egger’s test, which are qualitative and quantitative methods, respectively. In the Egger’s test, P < 0.05 indicates a statistically significant publication bias. In our study, statistical analyses were performed using R (Version 4.0.3) and STATA 12.

## 3 Results

### 3.1 Study selection

**Figure 1:**
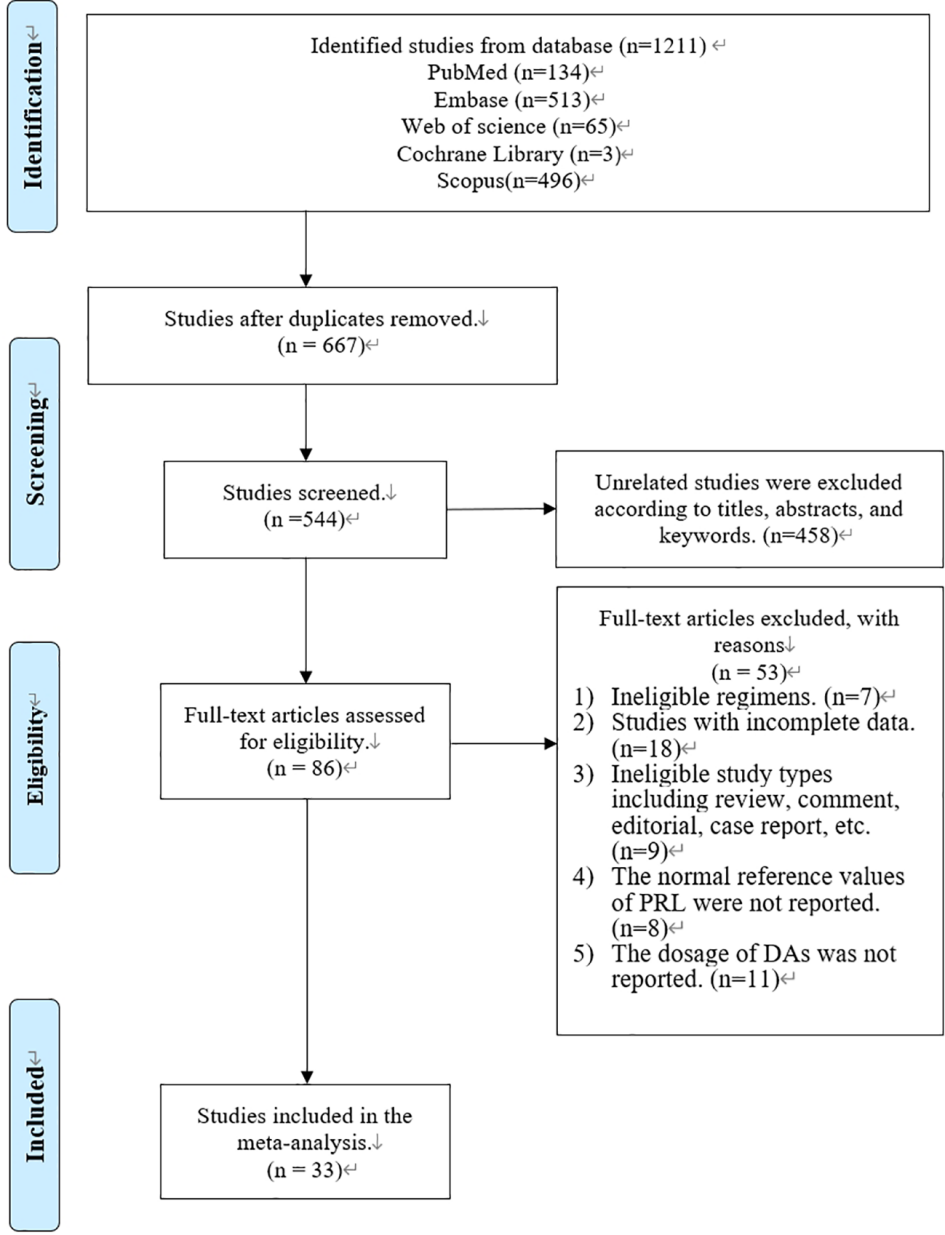
Flow diagram of the studies included in this systematic review and meta-analysis.

In total, 1,211 articles were retrieved from PubMed (n = 134), Embase (n = 513), Web of Science (n = 65), Cochrane Library (n = 3), and Scopus (n = 496) (**Figure 1**). A total of 667 articles were removed *via* a duplication check. The remaining 544 articles were screened according to the titles, abstracts, and keywords, and 458 of the articles were removed. Afterward, 86 articles were screened *via* a full-text check, and 53 of the articles were removed due to ineligible regimens (n = 7), incomplete data (n = 18), ineligible study types (n = 9), no reference values (n = 8), and no dosage information (n = 11). Finally, 33 studies were included in our systematic review and meta-analysis (**Figure 1**).

### 3.2 Study characteristics

In total, 33 studies with 827 patients and 178 arms were included in our systematic review and meta-analysis (9, 12–43). Among 178 arms, 59 arms referred to the efficacy of CV and 119 arms referred to safety.

In nine studies with double arms, one study was published in Spain (35), four were published in Italy (36–39), three were published in Switzerland (40, 41, 43), and one was published in Britain (42). Four studies compared CV and CAB (35–37, 39), and another five studies compared CV and BRC (38, 40–43). In tumor detection, three studies used magnetic resonance imaging (MRI) (35–37) and one study used both MRI and computed tomography (CT) (38). For serum PRL concentration measurements, seven studies used radioimmunoassay (RIA) (35, 38–43) and one study used immunoradiometric assay (IRMA) (36).

In 24 studies with a single arm, seven studies were retrospective (12–18) and 17 studies were prospective (9, 19–34). In the retrospective studies, for tumor detection, two studies used MRI (12, 16), one study used CT (15), and three studies used both MRI and CT (14, 17, 18). For serum PRL concentration measurements, six studies used RIA (12, 14–18) and one study used both IRMA and immunochemilu-minometric assay (ICMA) (13). In prospective studies, for tumor detection, one study used MRI (28) and four studies used both MRI and CT (19, 20, 22, 31). For serum PRL measurements, most studies used RIA, except for the studies by Vilar et al. (21) and Crottaz et al. (23).

The drug dosage and duration information are listed in **Tables S1**, **S2**. Detailed information is shown in **Tables 1**, **2**. For nine studies with double arms, one study included a first week dose of 25–50 µg/day and second week maintenance of 75 µg/day; one study included doses ranging from 25 to 75 µg/day and 150 µg/day after 12 weeks; one study included doses ranging from 25 to 75 µg/day; two studies included maintenance at 75 µg/day; three studies included doses ranging from 75 to 600 µg/day; and one study included a dose of 25 µg/day in the first week, 100 µg/day in the second week, and a maximum dose not to exceed 200 µg/day. In summary, the minimum doses ranged from 25 to 75 µg/day, with final doses not exceeding 600 µg/day. For 24 studies with a single arm, these single-arm trials were unstable, with drug doses below 600 µg/day (except for one study), and 75–300 µg/day was a more common dose.

**Table 1:** Characteristics and Cochrane risk of bias of included studies with double arms.

Year-First Author	Region	Study type	Cause of hyperprolactinemia	Drug (1)	Drug (2)	No. patients	Age (years)	Male/ Female	Tumor detection	PRL measurement methods	Initial PRL concentration (ng/ml)	Tumor reduction	PRL concentration normalization	Cochrane risk of bias
2000-De Luis	Spain	Double-blind, prospective, randomized controlled study	Microprolactinoma:8; idiopathic hyperprolactinemia:6; empty sella turcica syndrome:6	Quinagolide	Cabergoline	20	35.5 ± 8.6 (19–53)	2/18	MRI	RIA	CV: 89.2 ± 32; CAB: 81.3 ± 43.	\	CV:15/20; CAB:18/20.	**NNNNNY**
2000-Di Sarno	Italy	Prospective, controlled study	Microprolactinoma	Quinagolide	Cabergoline	23	23-54	2/21	MRI	IRMA	CV: 73 ± 7 (45–520); CAB: 129 ± 20.	CV: reduction>20%:5/23; reduction100%:4/23; CAB: reduction>20%:7/23; reduction:4%-40%:12/23; reduction100%:4/23.	CV:23/23; CAB:22/23.	YYYNNN
2000-Di Sarno	Italy	Prospective, controlled study	Macroprolactinoma	Quinagolide	Cabergoline	16	19-76	6/10	MRI	IRMA	CV: 99 ± 11 (114–3,083); CAB: 360 ± 210.	CV: reduction>20%: 4/16; reduction 100%: 2/16; CAB: reduction>20%:5/16; reduction:20%-70%:7/16; reduction100%:2/16	CV:14/16; CAB:14/16.	
2000-Colao	Italy	Prospective, controlled study	Prolactinoma	Quinagolide	Cabergoline	QUI: 5; CAB: 5	22-59	3/7	MR		263.7 ± 31.4	CV: reduction 30%: 1/5; reduction 50%: 1/5; reduction>80%: 3/5; CAB: reduction 30%:1/5; reduction>80%:4/5.	CV:3/5; CAB:4/5.	NYYNNY
1995-Colao	Italy	Prospective, controlled study	Macroprolactinoma	Quinagolide	Bromocriptine-LAR	QUI:16 BRC-LAR: 8	QUI:19-54 BRC-LAR:20-46	QUI:5/11 BRC-LAR:2/6	CT, MRI	RIA	CV: 615 ± 515 (250–2,050); BRC-LAR: 320 ± 171.7 (150–700)	\	CV:12/16; BRC-LAR:8/8	YYYUNN
1995-Colao	Italy	Prospective, controlled study	Macroprolactinoma	Quinagolide	Bromocriptine-SRO	QUI:16 BRC-SRO: 10	QUI:19-54 BRC-SRO:18-48	QUI:5/11 BRC-LAR:3/7	CT, MRI	RIA	CV: 615 ± 515 (250–2,050); BRC-SRO: 461 ± 329 (120–900)	\	CV:12/16; BRC-SRO:7/10.	
1994-Giusti	Italy	Prospective, randomized controlled study	Microprolactinoma:5; idiopathic hyperprolactinemia:6; Empty sella turcica syndrome:1	Quinagolide	Cabergoline	12	QUI:28.7 ± 5.2;CAB:31.7 ± 9.1	0/12	\	RIA	CV: 70.1 ± 7.6; CAB: 69.1 ± 8.2.	\	CV:6/12; CAB:10/12.	NYYUNN
1992-Lappohn	Switzerland	Prospective, randomized controlled study	Prolactinoma	Quinagolide	Bromocriptine	24	35 (22–24)	0/24	\	RIA	\	\	CV:8/12; BRC:8/12.	NNNNNN
1991-Van der Heijden	Switzerland	Double-blind, prospective, randomized controlled study	Prolactinoma	Quinagolide	Bromocriptine	47	18-47	0/47	\	RIA	\	\	CV:19/21; BRC:17/20.	NNNUNY
1991-Verhelst	UK	Double-blind, prospective, randomized controlled study	Macroprolactinoma:1; microprolactinoma:7; hyperprolactinemia:4	Quinagolide	Bromocriptine	12	19-56	2/10	\	RIA	\	\	CV:5/7; BRC:3/5.	NNNUNY
1990-Homburg	Switzerland	Double-blind, prospective, randomized controlled study	Macroprolactinoma:1; microprolactinoma:11; hyperprolactinemia:10	Quinagolide	Bromocriptine	22	33 (22–45)	0/22	\	RIA	138 (32–549)	\	CV:8/11; BRC:2/9.	NNNUNN

yr, year; PRL, prolactin; Bromocriptine-LAR, Bromocriptine-SRO: This is a different form of bromocriptine (BRC) with a long duration of action and slow absorption, suitable for injection (BRC-LAR) or oral administration (BRC-SRO); QUI, CV, Quinagolide; CAB, Cabergoline; BRC, Bromocriptine; MRI, magnetic resonance imaging; CT, computed tomography; RIA, radioimmunoassay; IRMA, immunoradiometric assay.

\: Unknown.

**Table 2:** Characteristics, Cochrane risk of bias, and Newcastle–Ottawa scale of included studies with single arm.

Year-First Author	Region	Study type	Cause of hyperprolactinemia	No. patients	Age (years)	Male/Female	Tumor detection	PRL measurement methods	Initial PRL concentration (µg/l)	PRL concentration normalization	Tumor reduction	Newcastle–Ottawa scale		
												Selection	Comparability	Exposure
2000-Schultz	USA	Single-blind, retrospective	No radiological evidence of pituitary tumor	17	33.3 ± 9.0 (1–53)	0/17	MRI	RIA	89.3 ± 73.2	14/17	\	★☆★☆	★★	★☆☆
2000-Schultz	USA	Single-blind, retrospective	Microprolactinomas	11	Unknown	0/11	MRI	RIA	223 ± 149	8/11	\			
2000-Schultz	USA	Single-blind, retrospective	Macroprolactinoma	12	Unknown	4/8	MRI	RIA	380 ± 331	8/12	\			
2000-Rohmer	French	Retrospective multicenter study	Macroprolactinoma:80;	107	Unknown	46/61	Unknown	IRMA or ICMA	748 ± 229 (2-14,700 )	47/107	Reduction<25%: 5/82;	★☆★☆	★★	☆★★
			Microprolactinoma:27								Reduction 25%-50%: 4/82;			
											Reduction>50%: 16/82.			
2000-Rohmer	French	Retrospective multicenter study	Prolactinoma	51	Unknown	Unknown	Unknown	IRMA or ICMA	577.58 ± 295.9	23/51	Reduction<25%: 4/51;			
											Reduction 25%-50%: 4/51;			
											Reduction>50%: 9/51.			
2000-Rohmer	French	Retrospective multicenter study	Prolactinoma	39	Unknown	Unknown	Unknown	IRMA or ICMA	748 ± 229 (2–14,700)	19/39	Reduction<25%: 1/31;			
											Reduction: 25%-50%:0/31;			
											Reduction>50%: 7/31.			
2000-Rohmer	French	Retrospective multicenter study	Prolactinoma	16	Unknown	Unknown	Unknown	IRMA or ICMA	748 ± 229 (2–14,700)	5/16	\			
1998-Colao	Italy	Retrospective	Macroprolactinoma: 5;	15	7–17	5/10	CT; MRI	RIA	Macroprolactinoma: 1,080 ± 267;	5/15	Reduction 20%-30%:1/15;	★☆★☆	★★	☆★★
			Microprolactinoma: 10.						Microprolactinoma: 155 ± 38.		Reduction>30%: 2/15.			
1996-Colao	Italy	Retrospective	Macroprolactinoma: 13;	14	36-45	14/0	CT	RIA	464 ± 75.7	13/14	\	★☆★☆	★★	☆☆☆
			Microprolactinoma: 1.											
1996-Morange	France	Retrospective	Macroprolactinoma: 21;	28	31.9 ± 2.0	8/20	MRI	RIA	404 ± 180	11/28	Reduction: 25%-90%: 5/21.	★☆★☆	★★	☆★★
			Microprolactinoma: 7.											
1992-Brue	France	Retrospective	Macroprolactinoma: 21;	27	29 ± 9 (13–50)	9/18	CT; MRI	RIA	2,295 ± 562	12/24	Reduction 25%-50%: 4/21;	★☆★☆	★☆	☆★☆
			Microprolactinoma: 6.								Reduction>50%: 1/21.			
1992-Brue	Switzerland	Retrospective	Macroprolactinoma: 16;	21	36 ± 2	9/12	CT; MRI		469 ± 160	11/21	Reduction >40%: 11/21	★☆★☆	★☆	☆☆☆
			Microprolactinoma: 5.											
1994-Merola	Italy	Prospective study	Macroprolactinoma: 7;	24	19-44	3/21	CT; MRI	RIA	70-1,677	Intolerance patients:10/10;	Reduction 25%-40%: 5/7.	**Cochrane risk of bias**		
			Microprolactinoma: 10;							Resistance patients:11/14				
			Hyperprolactinemia: 7.									YYYUNN		
1994-Merola	Italy	Prospective study	Macroprolactinoma: 16;	40	30.2 ± 1.5 (20–60)	10/30	CT; MRI	RIA	Macroprolactinoma: 615.5 ± 128.8 (180–2,050);	31/40	\	YYYNNY		
			Microprolactinoma: 14;						Microprolactinoma: 157.2 ± 14.5 (103–300);					
			Hyperprolactinemia: 10.						Hyperprolactinemia: 96.8 ± 10.1 (60–155).					
1994-Vilar	UK	Open-label, prospective study	Macroprolactinoma: 5;	12	Unknown	1/11	Unknown		Unknown	Intolerance patients:2/6;	\	YYYNNY		
			Microprolactinoma: 7.							Resistance patients:4/6				
1993-Kvistborg	Switzerland	Open-label, prospective study	Macroprolactinoma	16	45 (20–64)	9/7	CT; MRI	RIA	Unknown	12/16	Reduction>25%: 12/16.	YYYNNY		
1991-Crottaz	Switzerland	Prospective study	Macroprolactinoma	7	34-60	3/4			Unknown	4/7	\	YYYNNN		
1991-Barnett	UK	Prospective study	Macroprolactinoma	12	28-64	4/8	CT	RIA	Unknown	7/12	\	YYYNNN		
1991-Van der Lely	Switzerland	Prospective study	Macroprolactinoma	20	23-61	8/12	Unknown	RIA	Unknown	13/20	\	YYYNNY		
1991-Duranteau	France	Prospective study	Prolactinoma	7	22-74	1/6	Unknown	RIA	2,307 ± 518	2/7	\	YYYNNN		
1991-Shoham	UK	Prospective study	Prolactinoma	20	33.5 ± 6.6(23-45)	0/20	Unknown	RIA	66 (33–330)	14/20	\	YYYNNY		
1991-Rasmussen	Switzerland	Double-blind prospective study	Prolactinoma	24	39 (22–48)	0/24	Unknown	RIA	84 (31–1,100)	24/24	\	YNNNNN		
1990-Vance	USA	Open-label, prospective study	Macroprolactinoma	27	40 (17–73)	8/19	MRI	RIA	398.5 (2,051.7 ± 1,077)	15/26	Reduction<25%: 11/26;	YYYNNY		
											Reduction>50%:2/26;			
											Reduction 25%-50%:8/26.			
1990-Serri	Canada	Single-blind, prospective study	Macroprolactinoma: 9	15	40 (22–72)	9/6	CT	RIA	Female: 777;	Female: 5/6	Reduction<25%: 2/7;	YYNNNN		
									Male: 853.	Male: 6/9	Reduction 25%-50%:3/7;			
											Reduction>50%: 2/7.			
1990-Van’T Verlaat	Switzerland	Prospective study	Macroprolactinoma	11	18-73	5/6	CT	RIA	97-10,000	5/11	\	YYYNNN		
1990-Khalfallah	France	Open-label, prospective study	Macroprolactinoma	9	31.9 (20–47)	6/3	CT; MRI	RIA	235-1,320	8/9	Reduction>50%: 4/9.	YYYNNN		
1989-Van der Heijden	Switzerland	Double-blind prospective study	Prolactinoma	41	18-49	0/41	CT	RIA	Unknown	29/41	\	NNNUNN		
1989-Vance	USA	Double-blind prospective study	Microprolactinoma: 11	26	Unknown	0/26	Unknown	RIA	12.9 ± 3.4	13/24	\	YNNNNY		
1988-Rasmussen	Switzerland	Double-blind prospective study	Prolactinoma	24	39 (22–48)	0/24	Unknown	RIA	68 (31–1,100)	17/24	\	NNNUNN		

yr, year; USA, The United States of America; UK, The United Kingdom; MRI, magnetic resonance imaging; CT, computed tomography; RIA, radioimmunoassay; IRMA, immunoradiometric assay; ICMA, immunochemilu-minometric assay. “★”, “★★”: If the conditions listed in the Newcastle-Ottawa scale are satisfied, then a star can be assigned. \: Unknown.

### 3.3 The efficacy and safety of quinagolide

#### 3.3.1 Efficacy of quinagolide

##### 3.3.1.1 The efficacy of serum PRL: Prolactin. concentration normalization

**Figure 2:**
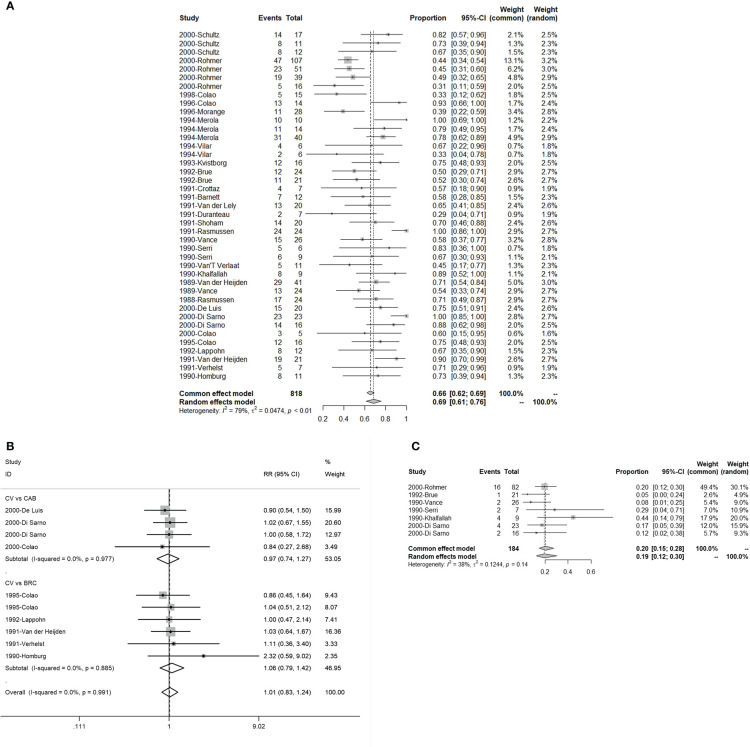
The efficacy of quinagolide (CV) in hyperprolactinemia and prolactinoma treatment. **(A)** The bulk efficacy of CV treatment in hyperprolactinemia; **(B)** The pooled risk ratios (RRs) of efficacy between CV vs. cabergoline (CAB) and CV vs. bromocriptine (BRC); **(C)** The efficacy of CV treatment in prolactinomas.

There were 24 studies with 41 single and 10 double arms that referred to the efficacy of serum PRL concentration normalization. The pooled proportion of normalized PRL concentration was 69% (95% CI: 61%–76%) (**Figure 2A**). For different initial serum PRL concentrations, we divided the initial serum PRL concentration into three levels based on the guidelines, which indicated that two thresholds (250 and 500 ng/ml) reflected the situations of hyperprolactinemia (4). The pooled proportions of normalized PRL concentrations for patients with low (<250 ng/ml), moderate (250–500 ng/ml), and high (>500 ng/ml) PRL levels were 0.81 (95% CI: 0.71–0.90), 0.59 (95% CI: 0.44–0.75), and 0.55 (95% CI: 0.43–0.67), respectively (**Figure S1**). In terms of patients with different sizes of prolactinomas, the pooled proportion of normalized PRL concentrations for patients with microprolactinoma was 90% (95% CI: 68%–100%) and that for patients with macroprolactinoma was 76% (95% CI: 69%–84%) (**Figure S2**). In terms of different treatment lines, the pooled proportion of normalized PRL concentrations for first-line CV treatment was 89% (95% CI: 77%–100%) and that for second-line treatment CV (patients with CAB/BRC resistance or intolerance) was 56% (95% CI: 46%–67%) (**Figure S3**). When comparing CAB and BRC, the pooled RRs of the proportion of PRL normalization were 0.97 (95% CI: 0.74–1.27) and 1.06 (95% CI: 0.79–1.42), respectively (**Figure 2B**).

##### 3.3.1.2 The efficacy of tumor reduction (>50%)

**Figure 3:**
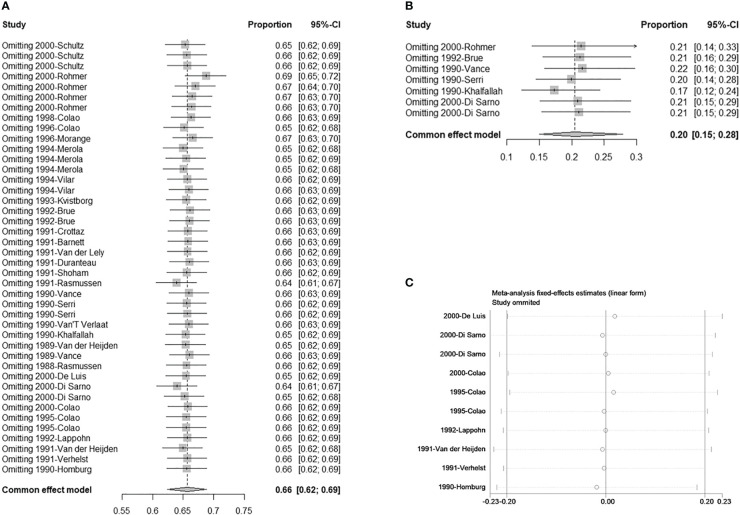
Sensitivity analysis. **(A)** Single-armed studies related to hyperprolactinemia treatment; **(B)** Single-armed studies related to prolactinoma treatment; **(C)** Double-armed studies.

**Figure 4:**
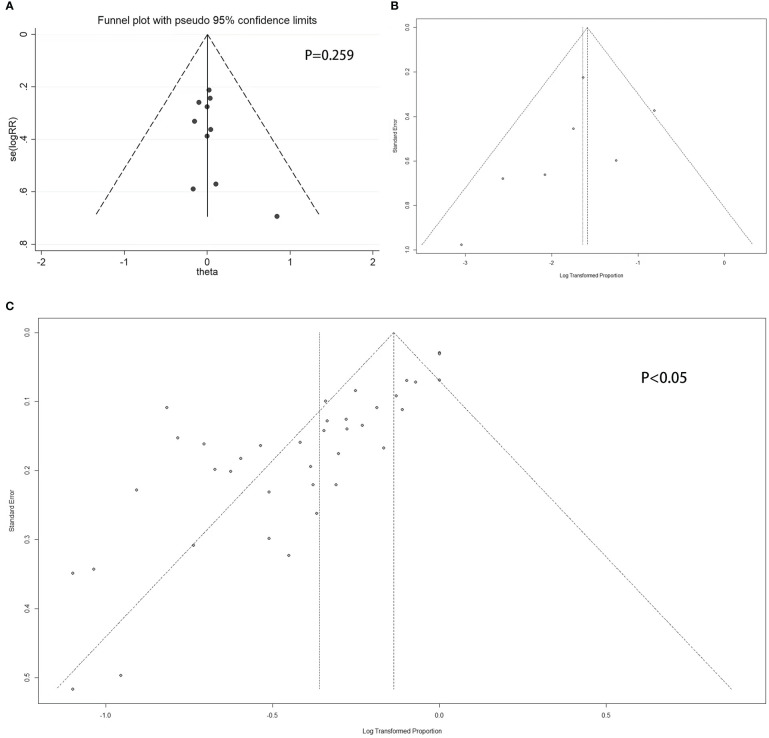
Funnel plot. **(A)** Double-armed studies; **(B)** Single-armed studies related to prolactinoma treatment; **(C)** Single-armed studies related to hyperprolactinemia treatment.

There were seven studies with eight double arms that referred to the efficacy of tumor reduction (>50%). However, the heterogeneity of the seven included studies was statistically significant (**Figure S4A**). The sensitivity analysis indicated that the study by Colao published in 2000 had a large impact on robustness (**Figure S4B**) (37). After removing this study, the heterogeneity of the remaining six studies was not significant (**Figure 2C**). The sensitivity analysis showed that the included studies were robust (**Figure 3B**). Moreover, a funnel plot did not find a potential publication bias (**Figure 4B**). The pooled proportion of tumor reduction (>50%) was 20% (95% CI: 15%–28%) (**Figure 2C**).

#### 3.3.2 Safety of quinagolide

**Figure 5:**
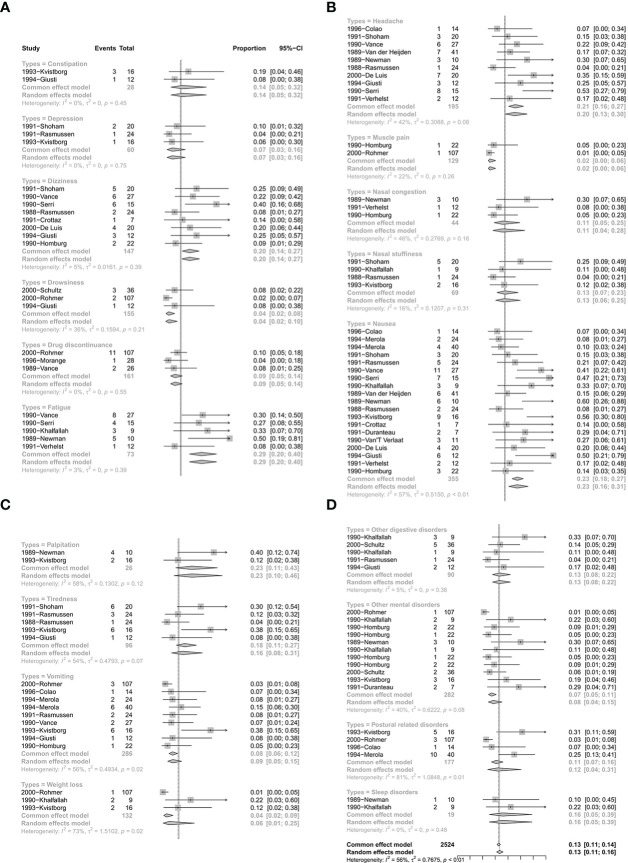
The safety of quinagolide treatment. **(A)** The pooled proportion of constipation, depression, dizziness, drowsiness, drug discontinuance, fatigue; **(B)** The pooled proportion of headache, muscle pain, nasal congestion, nasal stuffiness, nausea; **(C)** The pooled proportion of palpitation, tiredness, vomiting, weight loss; **(D)** The pooled proportion of other digestive disorders, other mental disorders, postural disorders, sleep disorders.

There were 24 studies with 119 single arms that referred to the safety of CV. The pooled proportion of side effects under CV was 13% (95% CI: 11%–16%) (**Figure 5**). In terms of the different types of side effects, the pooled proportions of constipation, depression, dizziness, drowsiness, drug discontinuance, fatigue, headache, muscle pain, nasal congestion, nasal stuffiness, nausea, palpitation, tiredness, vomiting, weight loss, other digestive disorders, other mental disorders, postural related disorders, and sleep disorders were 14% (95% CI: 5%–32%), 7% (95% CI: 3%–16%), 20% (95% CI: 14%–27%), 4% (95% CI: 2%–10%), 9% (95% CI: 5%–14%), 29% (95% CI: 20%–40%), 20% (95% CI: 13%–30%), 2% (95% CI: 0%–6%), 11% (95% CI: 4%–28%), 13% (95% CI: 6%–25%), 23% (95% CI: 16%–31%), 23% (95% CI: 10%–46%), 16% (95% CI: 8%–31%), 9% (95% CI: 5%–15%), 6% (95% CI: 1%–25%), 13% (95% CI: 8%–22%), 8% (95% CI: 4%–15%), 12% (95% CI: 4%–31%), and 16% (95% CI: 5%–39%), respectively (**Figure 5**). Detailed results are shown in **Figure 5**.

### 3.4 Sensitivity analysis, stratified analysis, and meta-regression

The sensitivity analyses of single arms referring to normalized PRL concentrations and corrected tumor reduction, as well as double arms referring to CV vs. CAB and BRC, were robust (**Figures 3A–C**). In the stratified analysis and meta-regression, studies were divided according to treatment lines and sizes of prolactinomas. As shown in **Table 3**, only studies referring to second-line treatments had statistical significance (P = 0.0147 < 0.05), which indicated that they may be the source of the heterogeneity (**Table 3**).

**Table 3:** Meta-regression analysis.

	Estimate	se	zval	pval	ci.lb	ci.ub	sig
intrcpt	-0.1991	0.1397	-1.4248	0.1542	-0.473	0.0748	
Treatment Line	-0.134	0.1552	-0.8631	0.3881	-0.4382	0.1702	
(Mixed)							
Treatment Line	-0.4199	0.1721	-2.4399	0.0147	-0.7572	-0.0826	*
(Second)							
Treatment Line	-0.1113	0.1893	-0.5878	0.5567	-0.4824	0.2598	
(unknown)							
Size	0.1373	0.1948	0.7047	0.481	-0.2445	0.5191	
(microprolactinoma)							
Size	0.0735	0.122	0.6023	0.547	-0.1657	0.3127	
(Mixed)							
Size	0.145	0.2569	0.5645	0.5724	-0.3585	0.6486	
(No evidence)							
Size	0.1152	0.1537	0.7491	0.4538	-0.1861	0.4164	
(unknown)							

se, standard error; zval, z value; pval, p value; ci.lb., lower bounds of 95% confidence interval; ci.ub, upper bounds of 95% confidence interval.

“*”: If P < 0.05 indicates a statistically significance.

### 3.5 Publication bias

For double-armed studies concerning the efficacy of PRL concentration normalization, the funnel plot did not show a potential publication bias, with a non-statistically significant Egger’s test result (P = 0.259 > 0.05) (**Figure 4A**). For studies related to the efficacy of tumor reduction (>50%), the funnel plot did not show a potential publication bias (**Figure 4B**), whereas the Egger’s test was not performed due to the small number of studies. For single-armed studies concerning the efficacy of PRL concentration normalization, a funnel plot showed the potential of publication bias, with statistically significant Egger’s test results (P < 0.05) (**Figure 4C**).

## 4 Discussion

In our results, the pooled proportion of normalized PRL concentrations was 69% under CV treatment, which is a relatively satisfactory value. The efficacy of CV is affected by the size of prolactinomas that were initially detected, the treatment line, and the initial serum PRL concentration. When the initial serum PRL concentration was more than 500 ng/ml or larger tumors were detected at the time of diagnosis, the efficacy was significantly reduced (**Figures S1**, **S2**). Likewise, the use of CV as a first-line treatment resulted in better efficacy (**Figure S3**). Compared with CAB and BRC, the pooled RRs of the proportion of PRL normalization were 0.97 and 1.06 when compared with CAB and BRC, respectively (**Figure 2B**). These results indicate that CV is not less effective than CAB and BRC in treating hyperprolactinemia.

For tumor shrinkage, the pooled proportion of more than 50% tumor shrinkage after CV treatment was 20% (**Figure 2C**), which is less effective than CAB and BRC, wherein these treatments had overall therapeutic outcomes that were reported to be more than 20% and 30%, respectively (6).

When concerning the adverse effects of CV treatment, dizziness (20%), fatigue (29%), headache (20%), nausea (23%), and palpitations (23%) were more likely to occur (pooled proportion ≥20%). Although multiple reports in the literature have confirmed that CAB seems to be safe at the doses that were employed in hyperprolactinemic patients, which is still unsettled. The incidence of asymptomatic tricuspid regurgitation is higher when detected *via* systematic echocardiography (44–46). It has been reported in the literature that echocardiography is recommended in patients with hyperprolactinemia who are under CAB therapy (46). This undoubtedly increases the difficulty of patient compliance and the costs of treatment. To date, there are no reports of cardiac valve disease associated with CV in the treatment of hyperprolactinemia.

For the robustness of our study, the heterogeneity was statistically significant (**Figures 2A**, **S4A**). We then performed a sensitivity analysis, Egger’s test, and funnel plots to evaluate and attempt to determine the source of the heterogeneity. In the sensitivity analysis of studies related to tumor shrinkage, we found that the study by Colao published in 2000 was relatively more sensitive because the pooled proportion was changed from 32% (95% CI: 25%–41%) to 20% (95% CI: 15%–28%) (**Figures S4B**, **3B**). After removing this study, the heterogeneity was significantly reduced, with the value changing from 80% I^2^ (P < 0.01) to 38% I^2^ (P = 0.14) (**Figures 2C**, **S4A**). However, the sensitivity analysis of the studies related to PRL normalization did not identify the source of the heterogeneity. We further performed a meta-regression analysis to try to verify whether the basic information (including treatment line and tumor size) reduced the robustness. In the results of the meta-regression, studies related to second-line CV treatment may be the source of the heterogeneity (**Table 3**). Subsequently, the publication bias was evaluated *via* a funnel plot and Egger’s test. The results showed a publication bias in the studies with a single arm.

Although our systematic review and meta-analysis showed that the overall success rate of PRL normalization after CV was 69%, it is worth mentioning that the current definition of remission is that the serum PRL concentration is normalized and persistent for more than 2 years. So, the data on CV may lack accuracy. To better evaluate the efficacy and safety of CV, more CV-related studies with more than 2 years of follow-up are needed.

Currently, there are many feasible options for hyperprolactinemia and prolactinoma treatments, including surgery, radiotherapy, and DAs. Surgery and radiotherapy are relatively difficult for patients to accept due to their disadvantages, such as considerable trauma and obvious side effects. Hence, DAs represent a first-line treatment. The sole reliance on drugs to treat tumors is also the future trend of tumor prevention and treatment. CV is not less effective than CAB and BRC in treating hyperprolactinemia, and the side effects were not significant. We may consider CV as an alternative first-line or rescue treatment.

There were some limitations in our study. First, due to the fact that the common DAs that are used for hyperprolactinemia and prolactinoma treatments in recent years are CAB and BRC, only a few studies have reported on the efficacy of CV in hyperprolactinemia and prolactinoma treatments; hence, CV-related studies have mostly been published before 2000 without enough follow-up time. Therefore, the overall quality of the included studies was low. There are not enough data to evaluate the long-term remission rate and risk of recurrence after CV treatment. Second, most of the included patients in these studies were from Europe, and it is unclear as to whether CV is suitable for people from continents other than Europe. Thus, we suggest that more CV–hyperprolactinemia-related studies with more than 2 years of follow-up and included patients from different areas around the world be performed.

## 5 Conclusion

Our study showed that CV is not less effective than CAB and BRC in treating hyperprolactinemia, and the side effects were not significant. More CV-related studies are also needed in the future.

## Data availability statement

The original contributions presented in the study are included in the article/**Supplementary Material**. Further inquiries can be directed to the corresponding author.

## Author contributions

YYZ wrote this article. YZZ and QLH processed the data. YYZ and QLH contributed equally. MHL reviewed all the work, and other authors participated in the whole work. All authors contributed to the article and approved the submitted version.

## Supplementary material

Supplementary Figure 1 The efficacy of quinagolide treatment in hyperprolactinemia. Subgroup analysis is based on different level of initial serum prolactin concentration.

Supplementary Figure 2 The efficacy of quinagolide treatment in hyperprolactinemia. Subgroup analysis is based on size of prolactinomas at diagnosis.

Supplementary Figure 3 The efficacy of quinagolide treatment in hyperprolactinemia. Subgroup analysis is based on treatment time of quinagolide.

Supplementary Figure 4 The forest plot and sensitivity analysis of studies related to tumor shrinkage before correction. **(A)** Forest plot; **(B)** Sensitivity analysis.
