# Supplementary figures and images for: The efficacy and safety of quinagolide in hyperprolactinemia treatment: A systematic review and meta-analysis
Source: Front Endocrinol (Lausanne). 2023 Jan 24;14:1027905. doi: 10.3389/fendo.2023.1027905 (PMC9902948; doi:10.3389/fendo.2023.1027905)

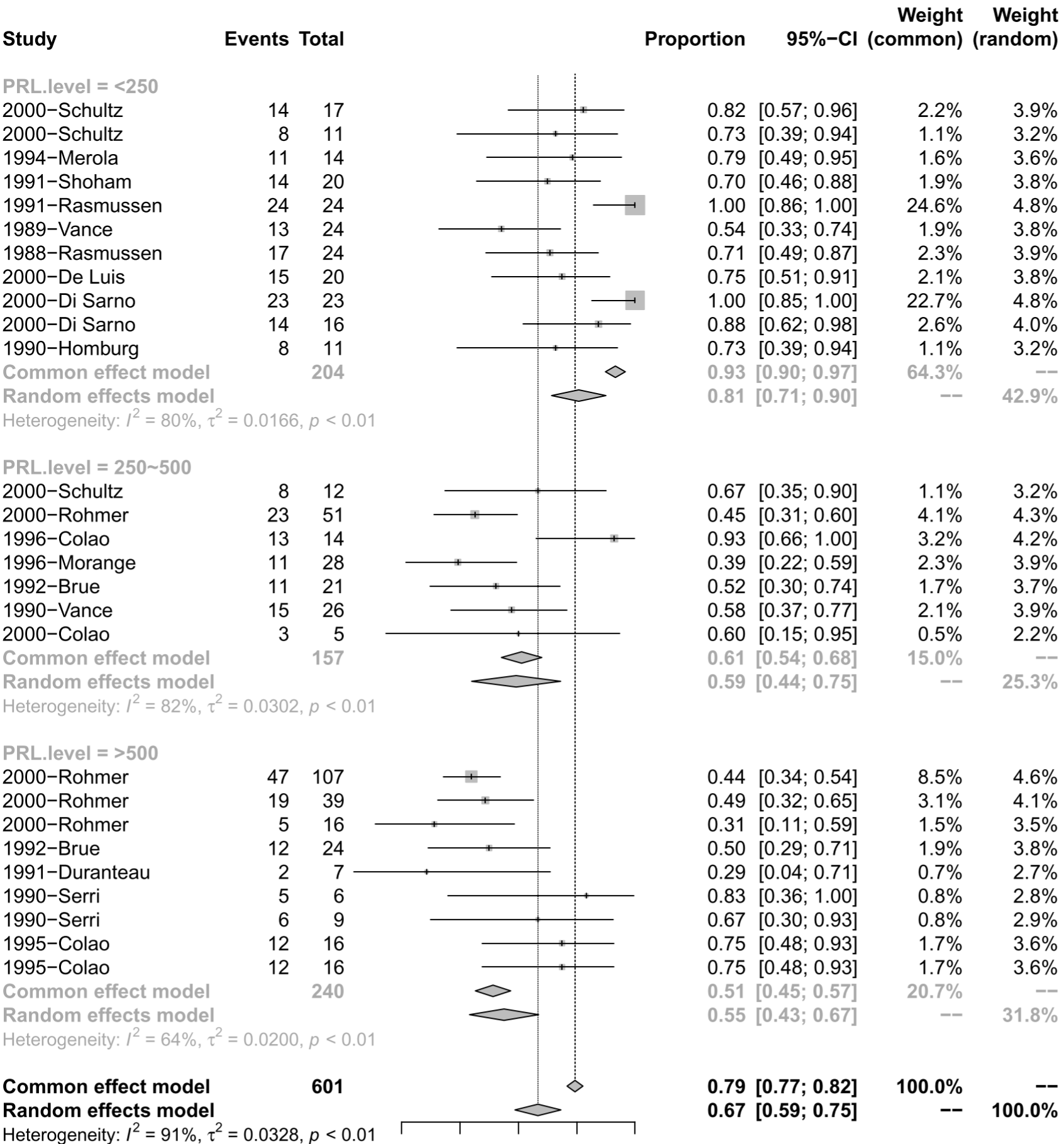

Supplement: Supplementary Figure 1 — The efficacy of quinagolide treatment in hyperprolactinemia. Subgroup analysis is based on different level of initial serum prolactin concentration. [file Image_1.png]

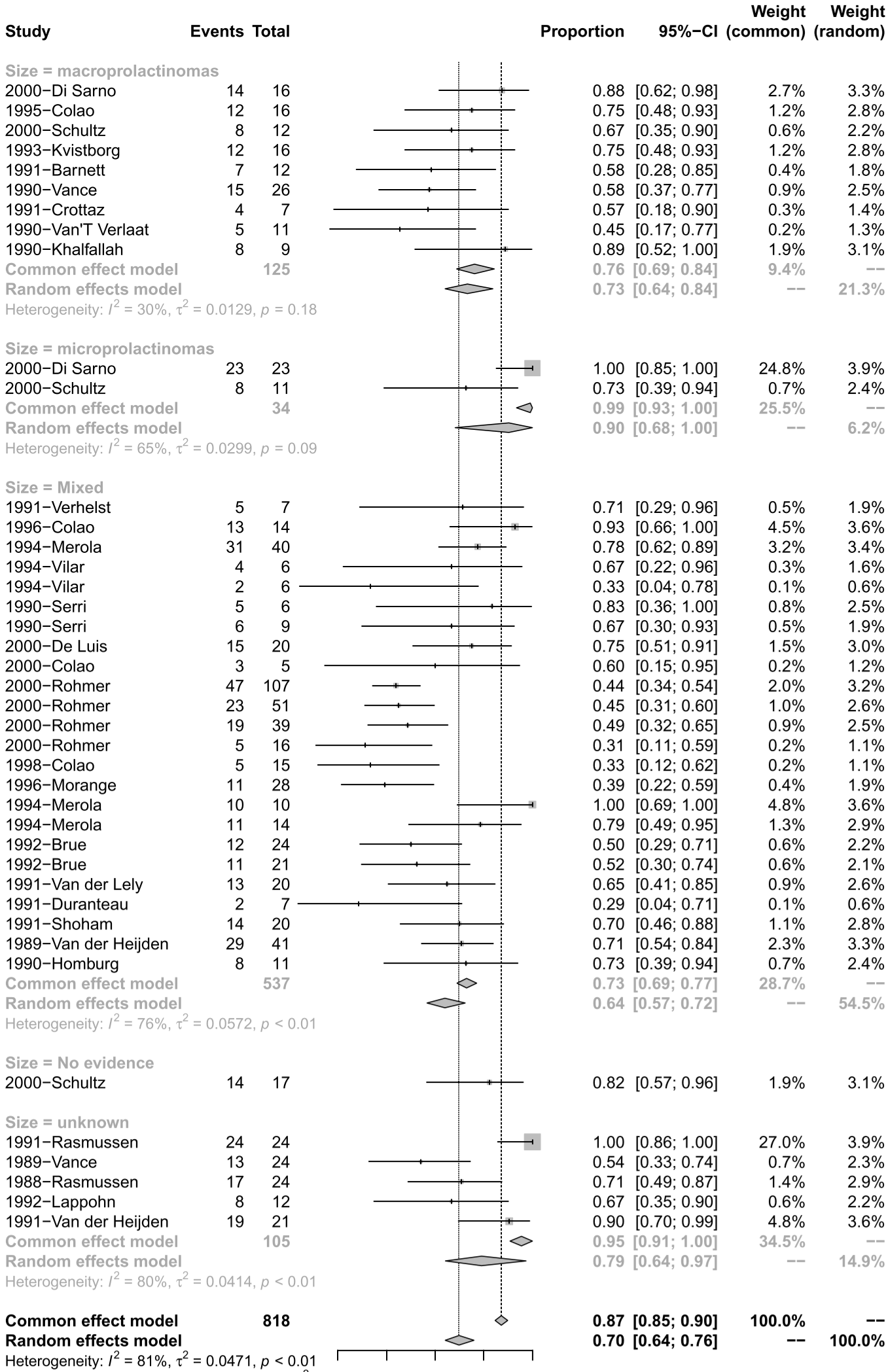

Supplement: Supplementary Figure 2 — The efficacy of quinagolide treatment in hyperprolactinemia. Subgroup analysis is based on size of prolactinomas at diagnosis. [file Image_2.png]

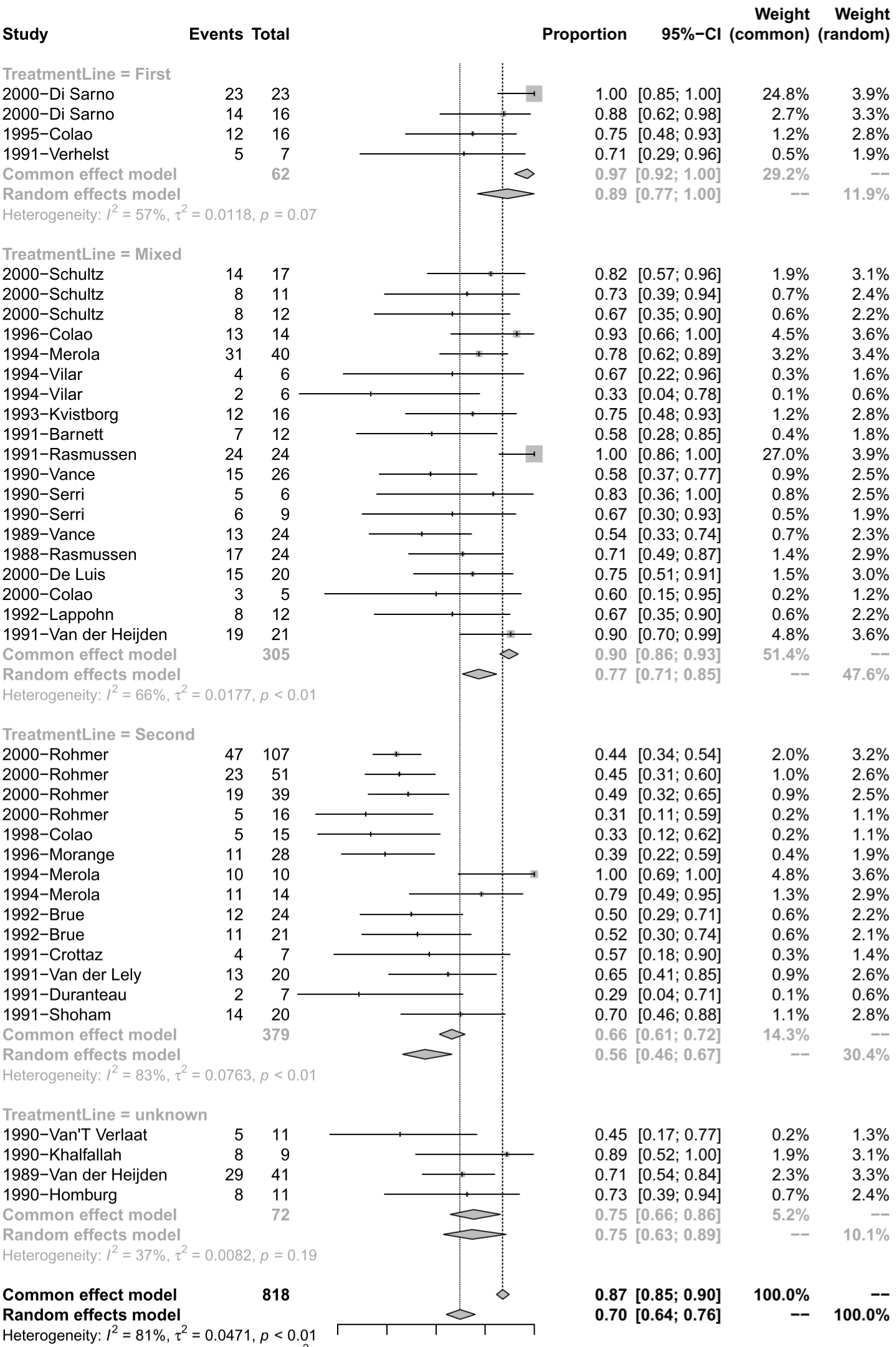

Supplement: Supplementary Figure 3 — The efficacy of quinagolide treatment in hyperprolactinemia. Subgroup analysis is based on treatment time of quinagolide. [file Image_3.png]

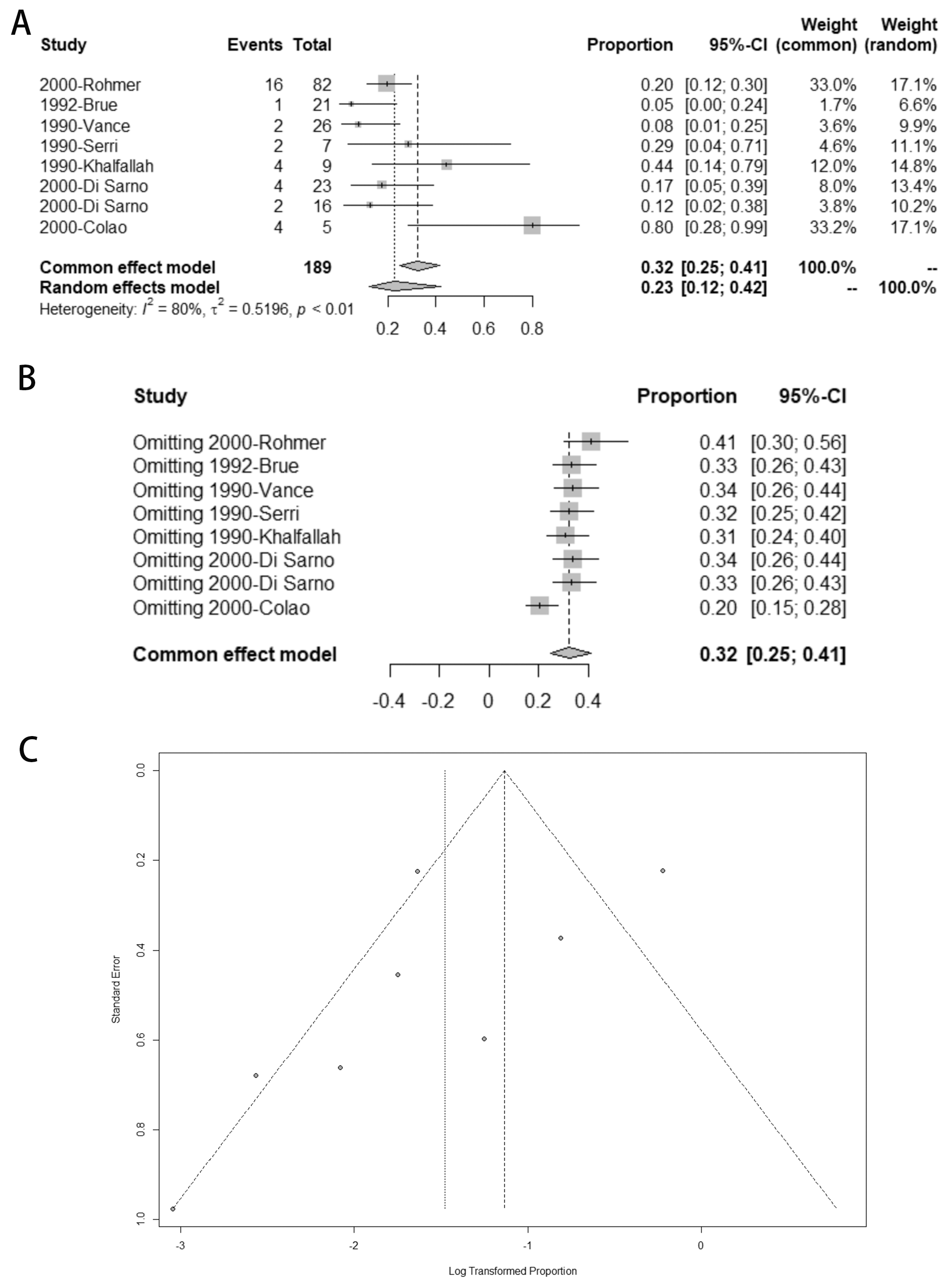

Supplement: Supplementary Figure 4 — The forest plot and sensitivity analysis of studies related to tumor shrinkage before correction. (A) Forest plot; (B) Sensitivity analysis. [file Image_4.png]
