# Supplementary material for: The efficacy and safety of quinagolide in hyperprolactinemia treatment: A systematic review and meta-analysis
Source: Front Endocrinol (Lausanne). 2023 Jan 24;14:1027905. doi: 10.3389/fendo.2023.1027905 (PMC9902948; doi:10.3389/fendo.2023.1027905)
Supplement: Supplementary file 5 [file Table_1.docx]

Table S1. Detail drug information of studies with double arms.

| **Year-First Author** | **Drug (1)** | **Duration (1) (Months)** | **Drug (2)** | **Duration (2) (Months)** |
| --- | --- | --- | --- | --- |
| 2000-De Luis | Quinagolide: once daily in a step-up regimen during the first week (from 25 ug to 50 ug/day); then the dose was increased to a maintenance dose of 75 ug/day. | 4 | Cabergoline: a starting dose of 250 ug twice during the first week and then at the dose of 500 ug twice weekly from week 2 to week 12. | 4 |
| 2000-Di Sarno | Quinagolide: The starting doses were 75 ug/day, subsequently increased up to 600 ug once daily. | 12 | Cabergoline: The starting doses were 500 ug/week, subsequently increased up to 1500 ug twice weekly. | 12 |
| 2000-Di Sarno | Quinagolide: The starting doses were 75 ug/day, subsequently increased up to 600 ug once daily. | 12 | Cabergoline: The starting doses were 500 ug/week, subsequently increased up to 1500 ug twice weekly. | 12 |
| 2000-Colao | Quinagolide: The doses ranged from 75 to 600 ug/day. | 12 | Cabergoline: The doses ranged from 500 to 3000 ug/week. | 12 |
| 1995-Colao | Quinagolide: daily dose of 75-600 ug. | 6-12 | BRC-LAR: monthly dose of 50-100 mg. | 6-24 |
| 1995-Colao | Quinagolide: daily dose of 75-600 ug. | 6-12 | BRC-SRO: daily dose of 5-20 mg. | 1-24 |
| 1994-Giusti | Quinagolide: 75 ug once daily. | 4 | Cabergoline: 500 ug twice weekly. | 4 |
| 1992-Lappohn | Quinagolide: The starting doses were 25 ug/day, subsequently increased up to 75ug once daily, according the PRL, 150 ug/d after 12 weeks. | 6 | Bromocriptine: The starting doses were 1.25 mg/d, subsequently increased up to 2.5 mg/d, according the PRL, 10 mg/d after 12 weeks. | 6 |
| 1991-Van der Heijden | Quinagolide: The starting doses were 25 ug/day, subsequently increased up to 75mg once daily. | 6 | Bromocriptine: The starting doses were 1250 ug/d, subsequently increased up to 2500 ug/d. | 6 |
| 1991-Verhelst | Quinagolide: initial dose of 25 ug/day and 100 ug/day after 1 week treatment, the maximum is 200 ug/day. | 6 | Quinagolide: initial dose of 1250 ug/day and 5000 ug/day after 1 week treatment, the maximum is 20000 ug/day. | 6 |
| 1990-Homburg | Quinagolide: once-daily doses of 75 ug. | 6 | Bromocriptine: daily dose of 5 mg. | 6 |

PRL: Prolactin ; BRC-LAR, BRC-SRO: This is a different form of bromocriptine (BRC) with a long duration of action and slow absorption, suitable for injection (BRC-LAR) or oral administration (BRC-SRO).
