# Supplementary material for: The efficacy and safety of quinagolide in hyperprolactinemia treatment: A systematic review and meta-analysis
Source: Front Endocrinol (Lausanne). 2023 Jan 24;14:1027905. doi: 10.3389/fendo.2023.1027905 (PMC9902948; doi:10.3389/fendo.2023.1027905)
Supplement: Supplementary file 6 [file Table_2.docx]

Table S2. Detail drug information of studies with single arm.

| **Year-First Author** | **Dosage** | **Duration (Months)** |  |
| --- | --- | --- | --- |
|  |  |  |  |
| 2000-Schultz | 136±80ug/day | 31.6 (2-72) |  |
| 2000-Schultz | 139±64 ug/day | 31.6 (2-72) |  |
| 2000-Schultz | 200±98 ug/day | 31.6 (2-72) |  |
| 2000-Rohmer | 255.4 ± 37.8 µg/d (75-750ug/d) | >12 |  |
| 1998-Colao | 75–600 ug/day | >3 |  |
| 1996-Colao | 75 ug/day for 15 days; afterwards it was increased up to 150-300 ug/day on the basis of PRL values | 6-24 |  |
| 1996-Morange | The starting dose of quinagolide (75ug/day) was rapidly increased to 150ug/day. At the 3rd month of treatment, according to the PRL controls and tolerance to the drug, the quinagolide daily dose given at bedtime was either continued (150µg) or increased up to 300- 450µg during the following months. | 43.9 ± 2.7 |  |
| 1992-Brue | 150-525 ug/day. |  |  |
| 1992-Brue | Daily dose of 25 ug was rapidly increased up to 100 ug and later to 500 ug. | 6 |  |
| 1994-Merola | Initial dose of 75 ug once daily.  Intolerance patients: increased to 300 ug, average 125 ug;  Resistance patients: increased to 0.6 mg, average 0.45 mg. | 3-12 |  |
|  |  |  |  |
| 1994-Merola | 75-600 ug/day. | 6-12 |  |
| 1994-Vilar | 75 ug nightly increasing incrementally to a maximum of 450ug. |  |  |
| 1993-Kvistborg | 25 ug/day. | 36 |  |
| 1991-Crottaz | 25-450 ug/day. | 4.25 |  |
| 1991-Barnett | 75 ug-1650 ug once daily. | 24 |  |
| 1991Van der Lely | Macroprolactinoma: 75-450 ug daily; PRL-secreting tumors: 75-300 ug daily. | 3;12 |  |
| 1991-Duranteau | Initial dose of 75 ug/day during the first week and then progressively increased up to 100 ug daily for 1 month. | 6 |  |
| 1991-Shoham | Daily dose of 75 to 150 ug | 3;24 |  |
| 1991-Rasmussen | Daily dose was increased by 25 ug every 4 weeks during the first 6 months, by 50 ug from month 6 to month 12, and by 100 ug (one patient) until month 21, with a maximum dose of 1300 ug daily. | 6;24 |  |
| 1990-Vance | Initial dose of 25ug/l and 75ug/l after 1 week treatment, the maximum is 400ug/l. | 2;3;6 |  |
| 1990-Serri | A daily dose of 25 ug, which was increased to 50 ug and then 75 ug by the end of week 1 and maintained at 75ug for a further 3 weeks Female:135ug(75-300ug); Male:192ug (75-300 ug). | 8.2 (6-12) |  |
| 1990-Van'T Verlaat | A dose of 75 ug once daily at first. The dose of CV 205-502 was gradually increased as long as the basal plasma  prolactin value remained elevated. | 2;18 |  |
| 1990-Khalfallah | Increased every 3 days from 25-75 ug. | 8-14 |  |
| 1989-Van der Heijden | Once-daily dose of 100 ug. | 3-13 |  |
| 1989-Vance | Initial dose ranged from 10-50 ug, and the dose at 12 weeks ranged from 30-90 ug. | 3 |  |
| 1988-Rasmussen | Once-daily doses of 50 to 150 ug. | 2;3;6 |  |

PRL: Prolactin; CV: Quinagolide.
